# Supplementary material for: EEG decoders track memory dynamics
Source: Nat Commun. 2024 Apr 6;15:2981. doi: 10.1038/s41467-024-46926-0 (PMC10998865; doi:10.1038/s41467-024-46926-0)
Supplement: Supplementary file 2 — Reporting Summary [file 41467_2024_46926_MOESM2_ESM.pdf]

nature portfolio

Corresponding author(s): NCOMMS-21-15247A, Michael Kahana

Last updated by author(s): Aug 4, 2023

Reporting Summary

Nature Portfolio wishes to improve the reproducibility of the work that we publish. This form provides structure for consistency and transparency in reporting. For further information on Nature Portfolio policies, see our Editorial Policies and the Editorial Policy Checklist.

Statistics

For all statistical analyses, confirm that the following items are present in the figure legend, table legend, main text, or Methods section.

n/a	Confirmed	
☐	☒	The exact sample size (n) for each experimental group/condition, given as a discrete number and unit of measurement
☐	☒	A statement on whether measurements were taken from distinct samples or whether the same sample was measured repeatedly
☐	☒	The statistical test(s) used AND whether they are one- or two-sided<br>*Only common tests should be described solely by name; describe more complex techniques in the Methods section.*
☒	☐	A description of all covariates tested
☐	☒	A description of any assumptions or corrections, such as tests of normality and adjustment for multiple comparisons
☐	☒	A full description of the statistical parameters including central tendency (e.g. means) or other basic estimates (e.g. regression coefficient) AND variation (e.g. standard deviation) or associated estimates of uncertainty (e.g. confidence intervals)
☐	☒	For null hypothesis testing, the test statistic (e.g. F, t, r) with confidence intervals, effect sizes, degrees of freedom and P value noted<br>*Give P values as exact values whenever suitable.*
☒	☐	For Bayesian analysis, information on the choice of priors and Markov chain Monte Carlo settings
☐	☒	For hierarchical and complex designs, identification of the appropriate level for tests and full reporting of outcomes
☐	☒	Estimates of effect sizes (e.g. Cohen's d, Pearson's r), indicating how they were calculated

Our web collection on statistics for biologists contains articles on many of the points above.

Software and code

Policy information about availability of computer code

Data collection	The experiment was written in Python using an in-house package (py-EPL; python experiment programming language). We recorded EEG using EGI's NetStation 4.3 system and BioSemi's ActiView (v7.05) system.
Data analysis	All data analyses (including post-processing, model fitting, and statistical analyses) used customized scripts written in Python (v2.7) and are available at https://github.com/pennmem/EEG-memory-dynamics-public. The core software packages used include MNE (v0.15), PTSA (v1.1.5), and sklearn (v0.19.1).

For manuscripts utilizing custom algorithms or software that are central to the research but not yet described in published literature, software must be made available to editors and reviewers. We strongly encourage code deposition in a community repository (e.g. GitHub). See the Nature Portfolio guidelines for submitting code & software for further information.

Data

Policy information about availability of data

All manuscripts must include a data availability statement. This statement should provide the following information, where applicable:

- Accession codes, unique identifiers, or web links for publicly available datasets
- A description of any restrictions on data availability
- For clinical datasets or third party data, please ensure that the statement adheres to our policy

Raw data in BIDS format has been published as OpenNeuro Dataset ds004395 (PEERS4 Experiment).

Research involving human participants, their data, or biological material

Policy information about studies with human participants or human data. See also policy information about sex, gender (identity/presentation), and sexual orientation and race, ethnicity and racism.

Reporting on sex and gender	Fifty-two out of a total of ninety-eight participants self-identified as Female. No sex- and gender-based analyses were performed. All participants gave consent for sharing of individual-level, gender- and identity- anonymized data.
Reporting on race, ethnicity, or other socially relevant groupings	*Please specify the socially constructed or socially relevant categorization variable(s) used in your manuscript and explain why they were used. Please note that such variables should not be used as proxies for other socially constructed/relevant variables (for example, race or ethnicity should not be used as a proxy for socioeconomic status).*<br>*Provide clear definitions of the relevant terms used, how they were provided (by the participants/respondents, the researchers, or third parties), and the method(s) used to classify people into the different categories (e.g. self-report, census or administrative data, social media data, etc.)*<br>*Please provide details about how you controlled for confounding variables in your analyses.*
Population characteristics	See "Research sample" below.
Recruitment	Participants were recruited through online and offline study advertisements. See "Research sample" below.
Ethics oversight	The study was approved by the Office of the Institutional Review Board at the University of Pennsylvania.

Note that full information on the approval of the study protocol must also be provided in the manuscript.

Field-specific reporting

Please select the one below that is the best fit for your research. If you are not sure, read the appropriate sections before making your selection.

☐ Life sciences ☒ Behavioural & social sciences ☐ Ecological, evolutionary & environmental sciences

For a reference copy of the document with all sections, see nature.com/documents/nr-reporting-summary-flat.pdf

Behavioural & social sciences study design

All studies must disclose on these points even when the disclosure is negative.

Study description	We collected quantitative data from a multi-session free recall task, including behavioral responses and EEG measures.
Research sample	Ninety-eight young adults (52 female, mean age=21.45, SD=3.06) recruited from among the students and staff at the University of Pennsylvania and neighboring institutions completed the study. This population was sampled primarily due to availability for multi-session commitment requiring 1.5 hr/session in-lab visit on separate days over an extended period of time.
Sampling strategy	We sought to recruit a sample of 100 participants who each completes the entire 24-session experiment to achieve the statistical power to look at both intra and inter-individual differences (e.g., Kahana et al., 2018 JEP:LMC). Across four years, 98 participants completed all 24 sessions.
Data collection	Participants visited for 24 sessions on separate days. In each session, participants completed a 24-list free-recall task presented on a computer screen and we recorded vocal free recall responses with a microphone. We recorded EEG with either a 129-channel EGI Geodesic Sensor Net in the Netstation acquisition environment or with a 128-channel BioSemi Active Two system. Participants were blind to the study hypothesis and were debriefed after pariticipation.
Timing	The study continuously recruited participants from Mar 2014 to May 2018.
Data exclusions	To ensure a diversity of successful and unsuccessful mnemonic events for binary classification, we excluded 10 participants prior to analysis whose recall rates were between 15% and 85% for fewer than 10 sessions.
Non-participation	Five participants withdrew due to reported EEG gel irritation, anxiety, panic attacks, and migraines during experiment.
Randomization	We collected repeated-measures from each participant undergoing an identical task, except stimuli order were randomized within-participant.

Reporting for specific materials, systems and methods

We require information from authors about some types of materials, experimental systems and methods used in many studies. Here, indicate whether each material, system or method listed is relevant to your study. If you are not sure if a list item applies to your research, read the appropriate section before selecting a response.

Materials & experimental systems

n/a	Involved in the study
☒	☐ Antibodies
☒	☐ Eukaryotic cell lines
☒	☐ Palaeontology and archaeology
☒	☐ Animals and other organisms
☒	☐ Clinical data
☒	☐ Dual use research of concern
☒	☐ Plants

Methods

n/a	Involved in the study
☒	☐ ChIP-seq
☒	☐ Flow cytometry
☒	☐ MRI-based neuroimaging
